# Editorial: Multimodality imaging in the assessment of ischemic chronic coronary syndrome

**DOI:** 10.3389/fcvm.2023.1146050

**Published:** 2023-04-11

**Authors:** Giuseppe Muscogiuri, Marco Guglielmo

**Affiliations:** ^1^ Department of Radiology, Istituto Auxologico Italiano IRCCS, San Luca Hospital, Milan, Italy; ^2^ School of Medicine, University of Milano-Bicocca, Milan, Italy; ^3^ Division of Heart and Lungs, Department of Cardiology, University Medical Center Utrecht, Utrecht University, Utrecht, Netherlands; ^4^ Department of Cardiology, Haga Teaching Hospital, The Hague, Netherlands

**Keywords:** CAD, multimodality, CMR, cardiac CT, ischemic heart, echocardiagraphy

**Editorial on the Research Topic** Multimodality imaging in the assessment of ischemic chronic coronary syndrome

Ischemic chronic coronary syndrome (ICCS) refers to a number of different conditions ranging from asymptomatic patients to ischemic cardiomyopathy (ICM) conditioning heart failure (1).

The diagnosis of ICCS is based on a combination of clinical history, physical examination, laboratory tests, and imaging tests (1).

The latter plays a critical role in the diagnostic work-up. Several imaging modalities are available, each with strengths and weaknesses. The choice of imaging modality depends on the patient's clinical presentation, the physician's preference, and equipment availability (1). The most commonly used noninvasive imaging modalities for the diagnosis of ICCS include echocardiography, nuclear medicine, cardiac magnetic resonance (CMR) and cardiac computed tomography angiography (CTCA).

In this special issue, El-Husseiny et al. use intraventricular pressure gradient (IVPG) as a noninvasive and sensitive approach to evaluate cardiac function. Specifically, their study aimed to examine IVPG's efficacy in predicting heart failure (HF) in a myocardial infarction (MI) rat model. The authors used transthoracic echocardiography (TTE) with color M-mode echocardiography to determine IVPG. Fifty healthy male rats were included, and MI was surgically induced in 35 of them. Based on echocardiographic parameters, the animals were categorized into two clusters: (heart failure positive, HF + or negative, HF-). After six months, the HF + group manifested the most pronounced echocardiographic, hemodynamic, anatomic, and histologic changes, and their basal IVPG was notably elevated compared to the HF- group. If confirmed by further research, IVPG obtained through echocardiography could be an auspicious noninvasive tool for detecting and predicting cardiac heart failure progression following chronic MI.

Islas et al. present interesting results regarding the prognostic role of epicardial adipose tissue (EAT) in patients suffering their first episode of MI and undergoing percutaneous coronary intervention (PCI). 41 patients (57.5 ± 10 years, 93% male) were enrolled. TTE and blood tests to measure galectin-3 (Gal-3) levels were performed within 24–48 h after PCI, while CMR was done 5–7 days after PCI. A clinical follow up of patients was performed after 1 and 5 years following MI.

Patients with EAT ≥ 4 mm measured on echocardiography showed lower left ventricular ejection fraction, abnormal global longitudinal strain, larger infarct size, and higher extracellular volume in the acute phase post-MI. Furthermore, the authors identified a cut-off value of EAT > 4 mm of thickness as an independent predictor of events 5 years after MI. EAT represents the visceral fat surrounding the heart and coronary arteries. In recent years, it has become the subject of increasing scientific investigation, with emerging evidence that it may be associated with the development of obstructive coronary artery disease and even acute coronary artery disease syndromes (2). 2D-Echocardiography does not allow for volumetric assessment of EAT, meaning it cannot accurately measure the total EAT volume like three-dimensional techniques such as CMR or CTCA. However, the study confirms that EAT thickness measured in 2D by a echocardiography can be used to predict patient outcomes and guide therapeutic interventions.

Focusing on CMR, Tat et al. showed that, in contrast to left ventricular ejection fraction, the extent and the location of late gadolinium enhancement (LGE) are independent predictors for the development of ventricular tachycardia. The study included 168 patients who underwent CMR imaging, either for ischemic cardiomyopathy (ICM) or non-ischemic cardiomyopathy (NICM). Of notice, the characteristics of LGE were found to have a more pronounced prognostic role for patients with NICM in comparison to subjects with ICM.

Unlike other methods, CMR has the great advantage of characterizing the myocardial tissue by identifying the presence of alterations, such as edema and focal and diffuse fibrosis (3). In particular, the LGE makes it possible to identify areas of focal fibrosis that often provide the substrate for threatening ventricular arrhythmias and sudden cardiac death. While CMR is a powerful diagnostic tool for evaluating ICCS, some potential disadvantages include costs, the use of contrast agents, and reduced availability.

Lala et al. wrote for this special issue an elegant mini-review on the multimodality approach in patients with ICC (3). The scope of the authors is to provide an overview of multimodality imaging for the diagnosis of chronic coronary syndrome in patients with new-onset HF and to suggest a diagnostic work-up based on the guidelines and data in the literature.

The algorithm of Lala et al. (3) for the diagnosis of new-onset and established HF includes the following steps (3):

1) performing a CTCA for rapid exclusion of CAD
2) if stenoses >50% are detected at CTCA, the next step is performing a stress CMR with LGE characterization. If CMR is unavailable, the authors propose a dobutamine stress echo as a second choice.
3) For advanced centers, Lala et al. suggest fractional flow reserve CT-based (FFR-CT) if >50% stenoses are identified with CTCA, followed by CMR with LGE tissue characterization.

Indeed, the diagnosis and management of ICCS often require a multimodality imaging approach that integrates information from various imaging techniques to comprehensively assess coronary anatomy, myocardial perfusion, and ventricular function (2, 4, 5).

By combining the information obtained from multiple imaging techniques, clinicians can provide patients with optimal care and improve their outcomes.

We believe that the application of artificial intelligence (AI) in the field of cardiac imaging will further improve the diagnostic workflows (4, 6).

By using AI algorithms to analyze imaging data, clinicians can obtain rapid, accurate, and detailed information about the cardiovascular system expedite correct diagnosis guide optimal treatment and improve prognosis.

In echocardiography, AI algorithms can improve the accuracy of measurements of left ventricular ejection fraction (LVEF) and other cardiac function parameters. AI algorithms can also be used to reduce the variability of measurements between different operators, leading to more consistent and accurate results (7).

AI algorithms can be used to analyze large CMR datasets to identify (8–11) areas of myocardial damage, quantify myocardial perfusion, and assess cardiac function. These algorithms can also be used to monitor changes in the cardiovascular system over time, providing clinicians with valuable information on disease progression and treatment response.

Given these premises, we expect a bright future for diagnosing ICCS using multimodality imaging.
